# Mining of prognosis-related genes in cervical squamous cell carcinoma immune microenvironment

**DOI:** 10.7717/peerj.9627

**Published:** 2020-08-24

**Authors:** Jiong Ma, Pu Cheng, Xuejun Chen, Chunxia Zhou, Wei Zheng

**Affiliations:** 1 Department of Gynecology, Second Affiliated Hospital, Zhejiang University School of Medicine, Hang Zhou, China; 2 Key Laboratory of Tumor Microenvironment and Immune Therapy of Zhejiang Province, Hang Zhou, China

**Keywords:** Cervical carcinoma, TCGA, Immune, Prognosis

## Abstract

**Purpose:**

The aim of this study was to explore the effective immune scoring method and mine the novel and potential immune microenvironment-related diagnostic and prognostic markers for cervical squamous cell carcinoma (CSSC).

**Materials and Methods:**

The Cancer Genome Atlas (TCGA) data was downloaded and multiple data analysis approaches were initially used to search for the immune-related scoring system on the basis of Estimation of STromal and Immune cells in MAlignant Tumour tissues using Expression data (ESTIMATE) algorithm. Afterwards, the representative genes in the gene modules correlated with immune-related scores based on ESTIMATE algorithm were further screened using Weighted Gene Co-expression Network Analysis (WGCNA) and network topology analysis. Gene functions were mined through enrichment analysis, followed by exploration of the correlation between these genes and immune checkpoint genes. Finally, survival analysis was applied to search for genes with significant association with overall survival and external database was employed for further validation.

**Results:**

The immune-related scores based on ESTIMATE algorithm was closely associated with other categories of scores, the HPV infection status, prognosis and the mutation levels of multiple CSCC-related genes (HLA and TP53). Eighteen new representative immune microenvironment-related genes were finally screened closely associated with patient prognosis and were further validated by the independent dataset GSE44001.

**Conclusion:**

Our present study suggested that the immune-related scores based on ESTIMATE algorithm can help to screen out novel immune-related diagnostic indicators, therapeutic targets and prognostic predictors in CSCC.

## Introduction

Cervical squamous cell carcinoma (CSCC) is one of the most common malignancies in female reproductive system, which severely threatens female health and life quality (Marth et al., 2018). CSCC is highly prevalent in developing countries, accounting for 60–90% of global cases (Chen et al., 2017). Radical hysterectomy is currently considered as the dominant therapy for early-stage cervical cancer (Gil-Moreno & Magrina, 2019; Uppal et al., 2019). With the popularization of cervical cancer screening, the therapeutic efficacy and prognosis of early-stage patients has been greatly improved (Altobelli et al., 2019; Ngo-Metzger & Adsul, 2019). Postoperative relapse and metastasis of CSCC remain the major causes of death in clinical practice (Alvarado-Cabrero et al., 2017; Nanthamongkolkul & Hanprasertpong, 2018). Patients with advanced-stage CSCC generally undergo adjuvant radiotherapy and/or chemotherapy; however, the therapeutic effect seems unsatisfactory (Angeles et al., 2019; Bosque, Cervantes-Bonilla & Palacios-Saucedo, 2018). At present, the International Federation of Gynaecology and Obstetrics (FIGO) staging classification is the major criterion for the prognostic prediction of patients with CSCC (Matsuo et al., 2019). Nevertheless, CSCC patients within similar clinical stage usually show diverse prognostic outcomes. Indeed, we now understand that the natural history of CSCC tumorigenesis is a continuous progress accompanied by a series of gene mutations over time. Based on this, CSCC was considered as a heterogenous collection of diseases, which were regarded as the major cause of anti-cancer treatment resistance and cancer relapse (Bachtiary et al., 2006; Kidd & Grigsby, 2008; Srivastava et al., 2019). As FIGO staging lacks heterogeneity of CSCC, clinical treatment decisions are now made depending on multiple factors including gene expression and mutation status other than traditional clinicopathological features. Thus, there is an urgent need to identify high-risk subgroups for individualized monitoring and optimized postoperative therapy in routine clinical practice.

Given the increasing evidence that various immune cells and inflammatory mediators are closely associated with the development of CSCC, tumor immune microenvironment is drawing accumulating attention nowadays (Piersma, 2011). The leukocytes, neutrophils, lymphocytes and macrophages directly contribute to the immune response, which could be easily and conveniently detected (Chen et al., 2019; Heintzelman, Lotan & Richards-Kortum, 2000; Lu et al., 2018; Rangel-Corona et al., 2011). In addition, several immune checkpoint biomarkers and cytokines have been identified to mediate the crosstalk between cancer cells and stromal microenvironment (De Nola et al., 2019; Otter et al., 2019). In the last decade, various studies have investigated the relationship between the prognosis of patients with primary CSCC and the immunological landscape through high-throughput quantitative measurements of cellular and molecular characteristics (Minion & Tewari, 2018; Punt et al., 2015). These studies revealed the great heterogeneity of the inflammatory/immune response in CSCC, which might determine to a large extent the final outcome of patients (Bachtiary et al., 2006). More recently, several researchers proposed a novel classification based on the immunological status of CSCC according to the ratio of different immune cells (such as monocyte/lymphocyte ratio or Th17/Treg ratio) in the tumor microenvironment, which might play a significant role in the accurate prediction of patient prognosis (Huang et al., 2019; Zhang et al., 2011). Unfortunately, almost none of the previous studies have reached clinical practice because of lacking the exploration from large sample data.

On this account, multiple immune scoring methods have been exploited using the expression data of immune-related genes in The Cancer Genome Atlas (TCGA) database which enable us to quantify the immune microenvironment status of a specific patient (Lee et al., 2012; Shen et al., 2018). For instance, the systemic immune-inflammation index (SII) established according to peripheral lymphocyte, neutrophil and platelet counts has been considered as a good indicator reflecting the local immune response and systemic inflammation (Fest et al., 2019). Moreover, SII has been confirmed to have remarkable association with the prognosis of numerous tumors, including non-small cell lung cancer (Guo et al., 2019), esophageal cancer (Ishibashi et al., 2018) and colorectal cancer (Xie et al., 2018). However, there have been only limited studies designed to develop an immune-related prognostic signature for CSCC up to now. Yang et al. (2019) established a prognostic immune signature for CSCC based on differential expression analysis and LASSO penalized Cox proportional hazards regression. However, like many other immune-related models (Cheng et al., 2016; Li et al., 2020; Lu et al., 2020), the variables were screened out based on single gene expression or immune cell proportion. Therefore, despite the predictive efficiency of these models, their relevance to tumor immune microenvironment is still need to be demonstrated. Moreover, among several existing immune-scoring systems, which one is most suitable for CSCC is waiting to be solved.

Earlier published work by Yoshihara group presents a new algorithm that uses the transcriptional profiles of cancer samples to infer the fraction of infiltrating stromal and immune cells, called ESTIMATE (Yoshihara et al., 2013). Importantly, the ESTIMATE method can be broadly applied across almost all human solid cancers. Thus, the ESTIMATE method is a powerful tool for oncologists to elucidate the complex roles of tumor microenvironment and explore potential solutions for tumor heterogeneity.

To this end, our present study was designed to explore the immune scoring method suitable for CSCC. In addition, the gene members in the scoring system were further analyzed by a series of bioinformatic means to mine the novel and potential immune microenvironment-related diagnostic and prognostic markers.

## Material and Methods

### Database sources and pre-processing

**Figure 1:**
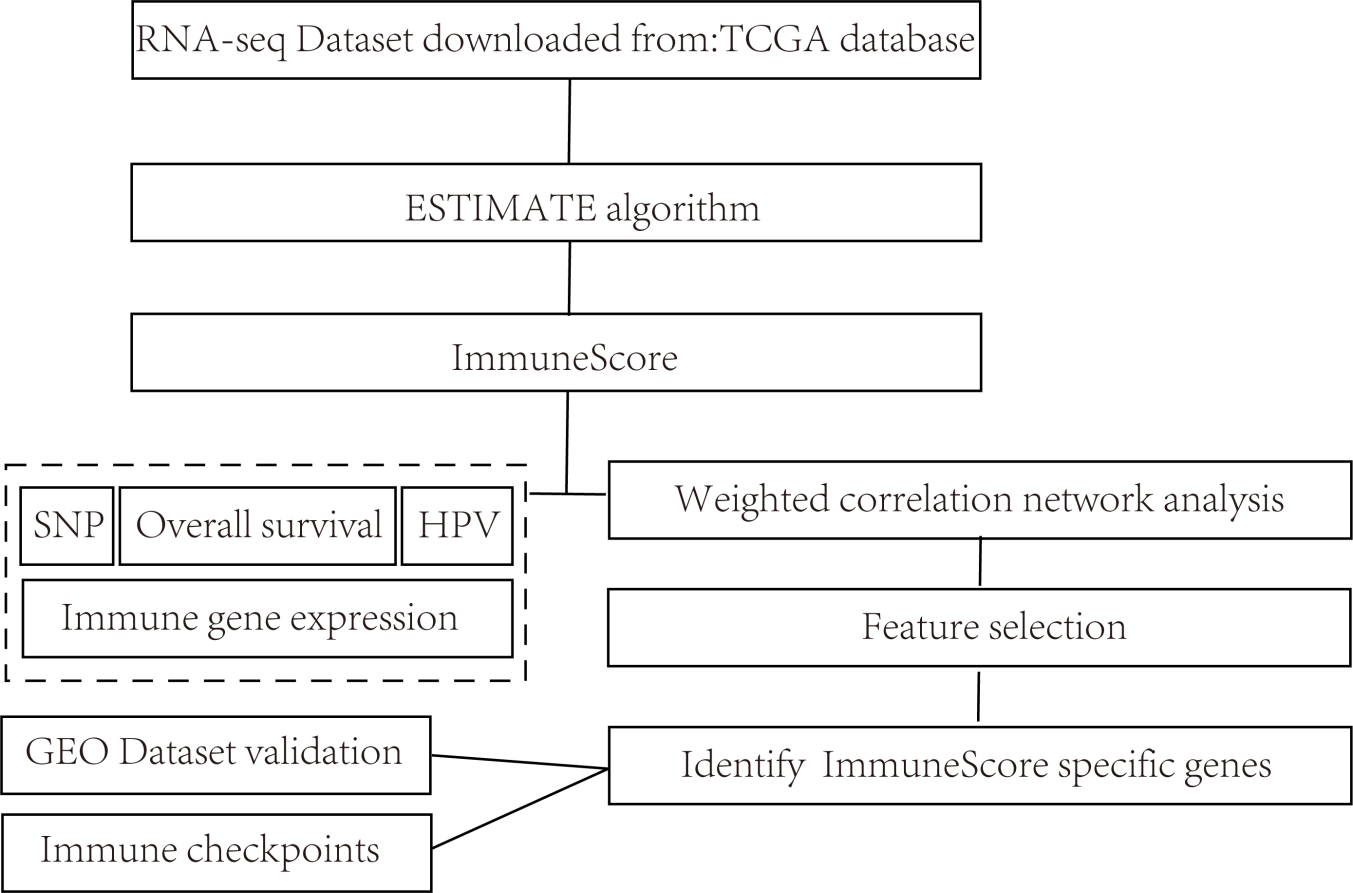
Flow diagram of methods for mining of prognostic immune-related genes in CSCC.

The overall flow diagram of present study was summarized in Fig. 1. The RNA-seq counts data, SNP data, and clinical follow-up information were downloaded from the TCGA database. The Reads PerKilobase Million (FPKM) data of RNA-Seq were transformed into Transcripts PerKilobase Million (TPM) expression profiles. In consistent with previous studies, 13 metagenes (shown in ImmuneScore.genes.ids.txt, Supplemental File) corresponded to various immunocyte types, reflecting the different immune functions.

### Computational methods of multiple immune scores and result determination

The scores of each sample in the 13 types of metagenes were calculated based on the log2-transformed expression of each gene member in the immune metagene (shown in immune.meta.score.txt, Supplemental File) (Safonov et al., 2017). TIMER (https://cistrome.shinyapps.io/timer/) database (immune.immu.score.txt, Supplemental File) was utilized to calculate the scores of each sample in the immunocyte infiltration (six categories in total) (Li et al., 2016). Moreover, the ImmuneScore (that represents the infiltration of immune cells in tumor tissue), StromalScore (that captures the presence of stroma in tumor tissue) and ESTIMATEScore (that infers tumor purity) of each sample (immune.est.score.txt, Supplemental File) were calculated by ESTIMATE function of R software package (Yoshihara et al., 2013). Detailed description of above 3 scores could be found on the website below: https://bioinformatics.mdanderson.org/public-software/estimate/. Finally, R software package MCPcounter was utilized for the calculation of the abundances of ten immune-related cell (eight categories of immune cells, endothelial cells and fibroblasts) populations in the tumor microenvironment (Supplemental File).

### Survival analysis

Patients were divided into several groups according to each specific parameter (including ImmuneScore, StromalScore, ESTIMATEScore and gene expression level). Afterwards, the association between the gene expression level (or level of ImmuneScore, StromalScore, ESTIMATEScore) and overall survival was analyzed by univariate Cox regression model.

### The construction of immune scores-related gene modules through WGCNA

To begin with, transcripts with over 75% TPM of >1 and median absolute deviation (MAD) of >median were chosen from the expression profile data of all the obtained samples. Hierarchical clustering for cluster analysis of the samples was also adopted. Subsequently, samples with a distance of over 80,000 were taken as the outlier samples for screening. Moreover, the distance between any two transcripts was calculated by Pearson correlation coefficient, the establishment of the distance between any two transcripts was performed by the R software package Weighted Gene Co-expression Network Analysis (WGCNA) (Xia et al., 2019), and the soft threshold was set as eight for the screening of the co-expression modules. The co-expression network has been suggested to conform to the scale-free network. In other words, the logarithm of node with the connectivity of k (log(k)) should be negatively correlated with the logarithm of the occurrence probability of the specific node (log(P(k))), and the correlation coefficient should be >0.85. Proper *β* value was selected in order to ensure the network as a scale-free network. The expression matrix was subsequently transformed into the adjacent matrix, and the latter was further transformed into the topological matrix for gene clustering based on Topological Overlap Matrix (TOM) utilizing the average-linkage hierarchical clustering method in accordance with the mixed dynamic shear tree standard. In addition, the gene number of each gene network module was set at least 30. The dynamic shear method was used to determine the gene module, followed by calculation of the eigengene value of each module in succession. Afterwards, clustering analysis was performed on the modules, in which, modules close to each other were merged into a new module, with re-set appropriate height, deepSplit and minModuleSize values. Finally, the association of the acquired gene modules with ImmuneScore, StromalScore and ESTIMATEScore were separately calculated, in order to explore the gene modules with high correlation for further research.

### Establishment of the gene interaction network and functional analysis

Genes were mapped into the String database (Szklarczyk et al., 2019). The gene-gene interactions were acquired at the score threshold of >0.4, followed by visualization using Cytoscape software (Shannon et al., 2003). Meanwhile, Kyoto Encyclopedia of Genes and Genomes (KEGG) and Gene Ontology (GO) enrichment analysis was performed by utilizing the clusterprofile R package (Xu et al., 2019) to examine the signaling pathways affected by these genes.

## Results

### The immune-related scores based on ESTIMATE algorithm is the most suitable immune scoring method for CSCC

**Figure 2:**
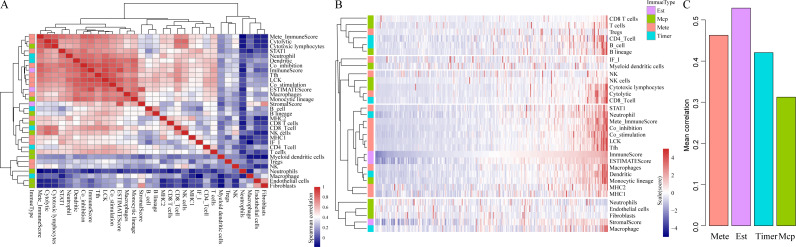
The correlations of immune-related scoring system based on ESTIMATE algorithm with other categories of scores among CSCC samples. (A) The correlations of various immune scoring systems among CSCC samples. Spearman correlation coefficients are shown color-coded to illustrate positive (red) or negative (green) associations. (B) The clustering heat maps of various types of scoring systems. (C) The relationships among immune scores according to four different algorithms. Mete, metagene immune score; Est, ESTIMATE immune score; Timer, Timer immune score; Mcp, MCPcounter immune score.

To be specific, we retrieved CSCC samples from the TCGA database and analyzed their scores in 23 types of scoring systems, including 13 types of metagenes scores, six types of immunocyte infiltration scores, three types of immune-related scores according to ESTIMATE algorithm (ImmuneScore, StromalScore and ESTIMATEScore) and 10 types of abundances of immune-related cell. In addition, Spearman’s correlation coefficient was used to calculate the correlations among these scoring systems (shown in Fig. 2). As shown in Fig. 2A, the average correlation between different types of immune-related scores was greater than 0.4. Among which, ImmuneScore (*R* = 0.59), Co_inhibition (*R* = 0.59) and LCK (*R* = 0.62) had the highest relevance with other immune scores. These findings showed that there were fine consistency and comparability between different immune scoring systems. The clustering heat maps of various types of scoring systems were shown in Fig. 2B, suggesting the great correlation among the scoring systems MHC1, MHC2, Monocytic lineage, Dendritic, Macrophages, ESTIMATEScore, ImmuneScore, Tfh, LCK, Co_stimulation, Co_inhibition, Mete_ImmuneScore, Neutrophil and STAT1. We further investigated the average correlation among immune scores according to four different algorithms. As shown in Fig. 2C, the immune-related scores calculated by the ESTIMATE algorithm harbored the highest average correlation with the other three algorithms, which is greater than 0.52 on average. These findings implicated that the immune-related scores based on ESTIMATE algorithm were the most representative immune scoring methods for CSCC.

**Figure 3:**
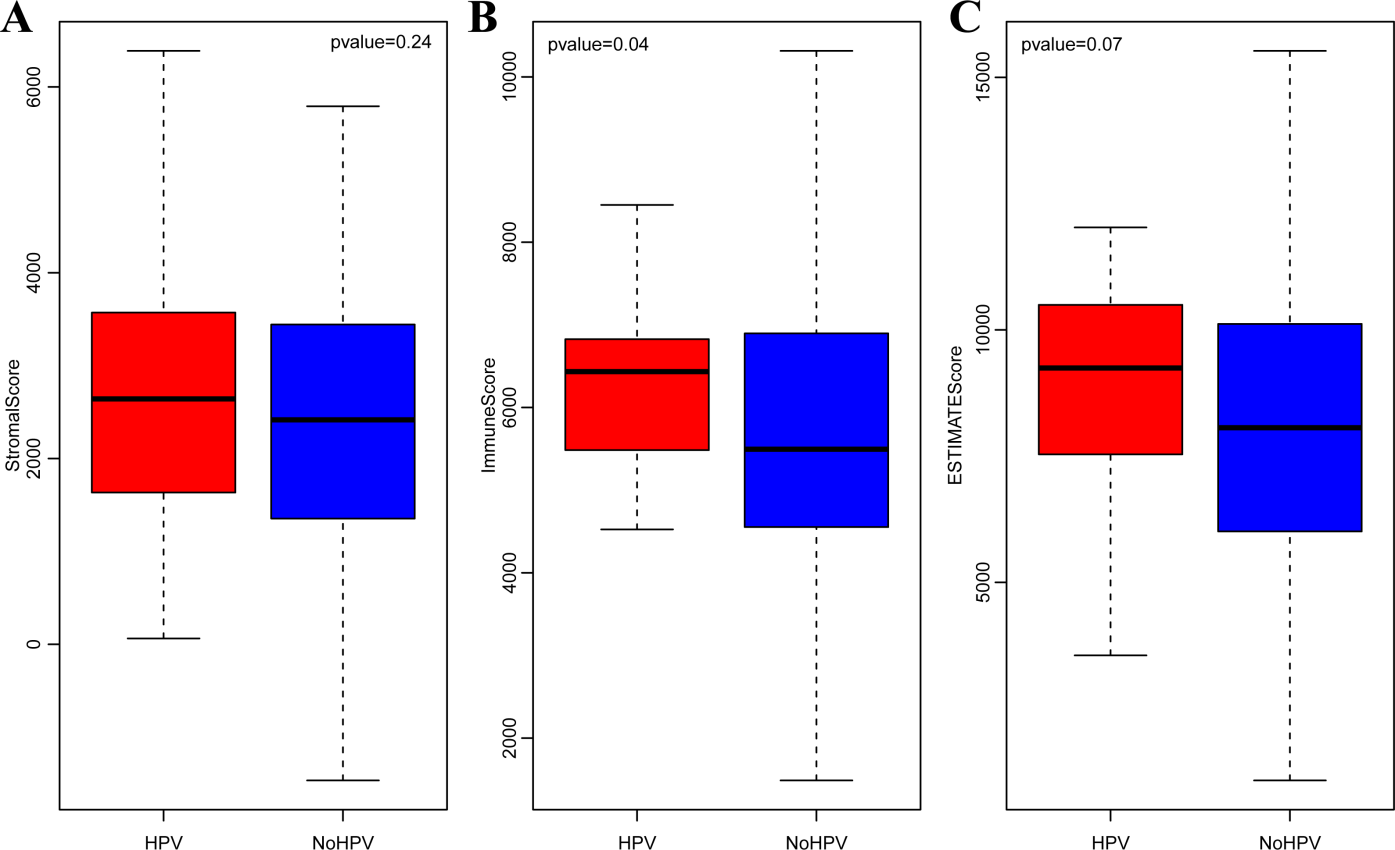
StromalScore (A), ImmuneScore (B) and ESTIMATEScore (C) distribution among CSCC patients with or without HPV infection.

It is widely accepted that HPV infection has a significant association with the occurrence and progression of CSCC (Ding et al., 2019). Therefore, we separately analyzed the ImmuneScore, StromalScore and ESTIMATEScore distribution among CSCC patients with or without HPV infection. As shown in Figs. 3A–3C, the three immune-related scores in CSCC with HPV infection were significantly higher than those without HPV infection. It should be noted that ImmuneScore was most significantly correlated with the infection status of HPV (*p* < 0.05).

**Figure 4:**
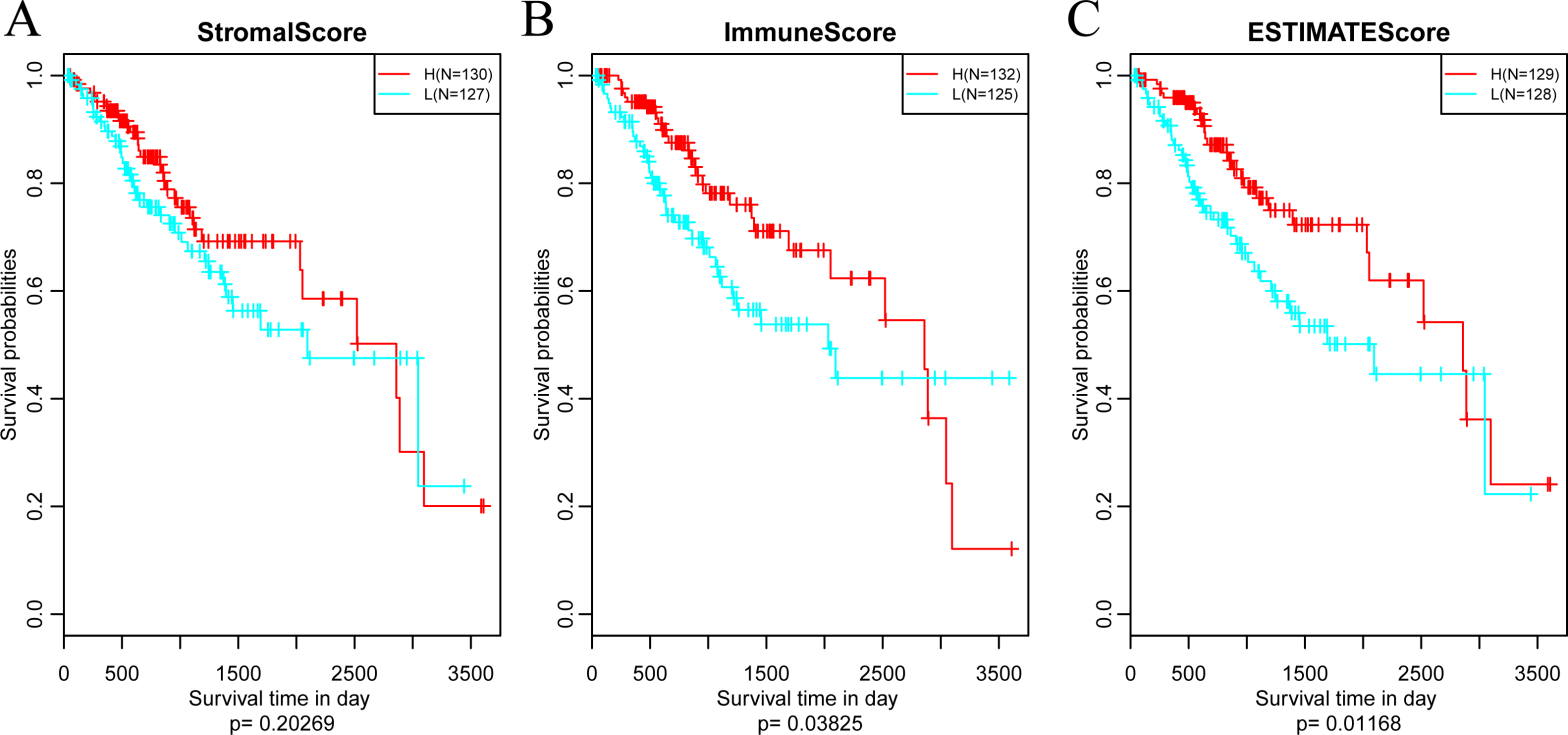
The relationships between levels of StromalScore (A), ImmuneScore (B) or ESTIMATEScore (C) and prognosis for CSCC patients. H, High immune score; L, Low immune score.

Subsequently, in order to investigate the association between the above three immune-related scores and prognosis, samples were sorted based on the median of scores of all samples. And then, prognostic difference was analyzed by Kaplan–Meier method (Zou, O’Malley & Mauri, 2007). As a result, the prognosis of samples in different groups was significantly different (shown in Fig. 4). And the five-year survival rate of samples with high ImmuneScore and ESTIMATEScore were significantly superior in comparison with those with low scores, suggesting that the three immune-related scores on the basis of ESTIMATE algorithm could be accepted as promising novel prognostic markers for CSCC.

**Figure 5:**
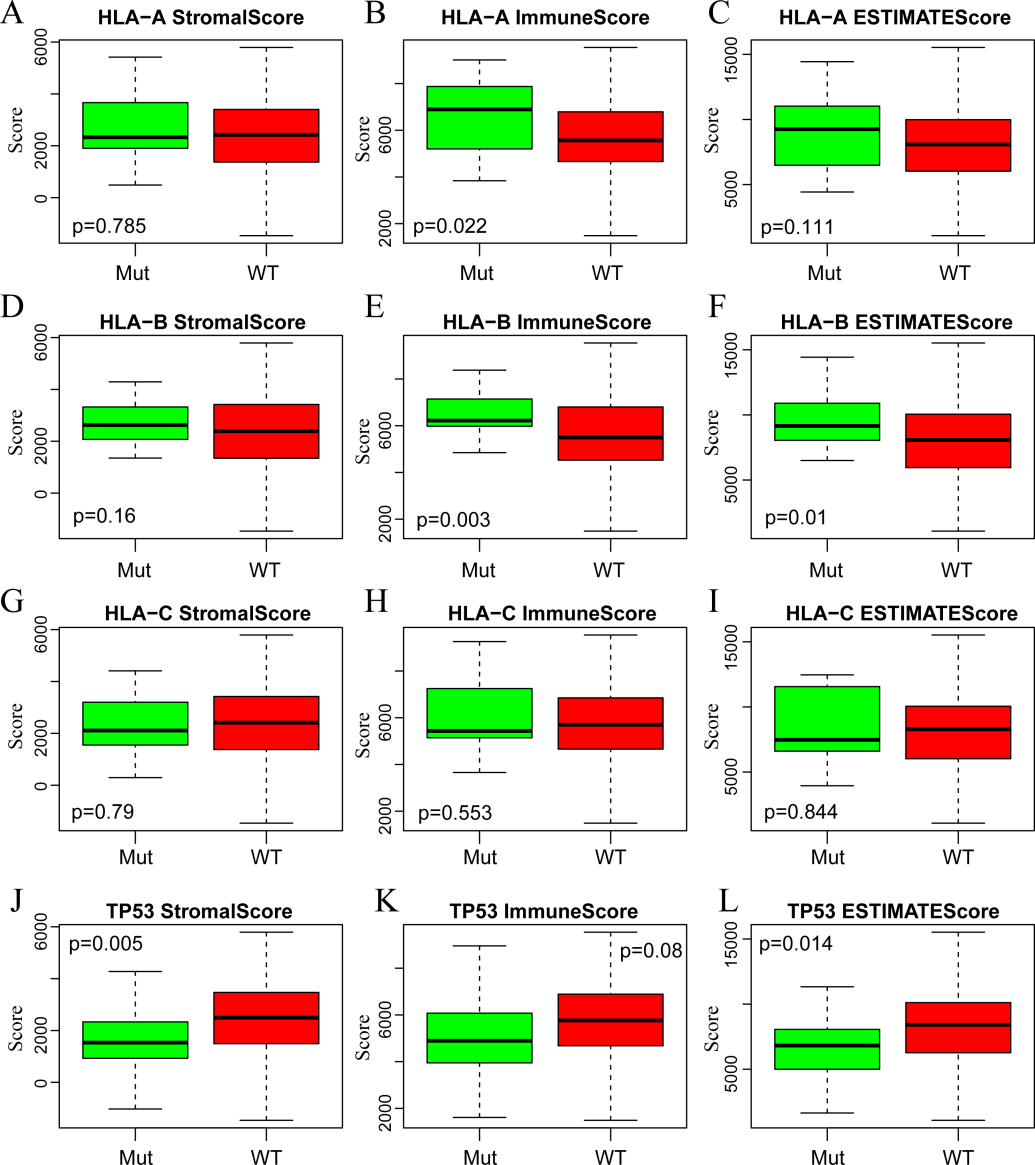
The correlations of immune-related scores based on ESTIMATE algorithm with gene mutations. The StromalScore (A, D, G, J), ImmuneScore (B, E, H, K) and ESTIMATEScore (C, F, I, L) were calculated respectively in HLA-A (A, B, C), HLA-B (D, E, F), HLA-C (G, H, I) and TP53 (J, K, L) mutation and non-mutation groups. Green represents the mutant group and red represents the wild type. Mut, Mutant; WT, Wild type.

A large number of somatic mutations of HLA genes have been reported in CSCC, strongly indicating that loss of function due to HLA mutations is tightly correlated with the immune escape of cancer cells (Xiao et al., 2013). It is of great significance for us to analyze the changes of HLA gene sequence in tumor patients. In addition, the mutation of TP53, a tumor suppressor gene, can induce unlimited proliferation and apoptosis resistance of tumor cells (Laprano et al., 2014; Li et al., 2015). Next, we focused on analyzing the associations of three immune-related scores with mutations of HLA and TP53. To this end, we extracted the mutation data of HLA-A, HLA-B, HLA-C and TP53 from the mutect-processed SNP database and then calculated the three immune-related scores based on ESTIMATE algorithm in HLA-A, HLA-B, HLA-C and TP53 mutation and non-mutation groups. As shown in Fig. 5, there was higher level of ImmuneScore in HLA-A and HLA-B mutation groups compared with wild-type groups, while there was also higher level of ESTIMATEScore in HLA-B mutation groups but lower level in TP53 mutation groups comparison with that in wild-type groups.

In summary, we demonstrated that the immune-related scores on the basis of ESTIMATE algorithm were the most proper immune scoring method for CSCC. Additionally, the co-expressed genes with remarkable correlation with these three immune-related scores might be considered as the representative genes in CSCC immune microenvironment, which could be further validated as potential prognostic markers and novel therapeutic targets of CSCC.

### Screening of the representative genes in the immune scores-related gene modules

**Figure 6:**
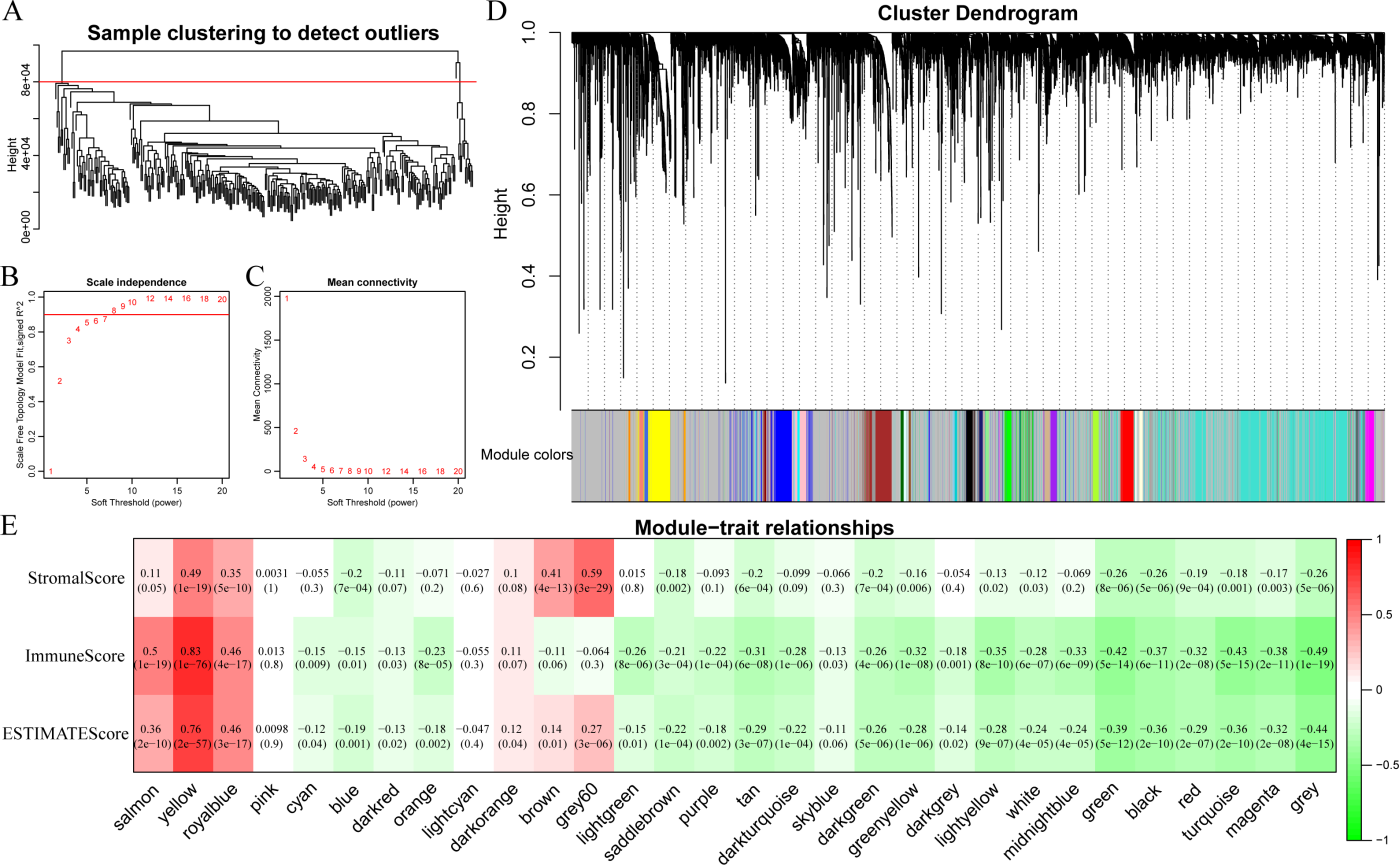
Immune scores-related gene modules mined through WGCNA. (A) Sample clustering analysis. (B, C) Analysis of network topology for various soft-thresholding powers. (D) Gene dendrogram and module colors. (E) Correlation between each module and three immune-related scores.

In this section, clustering analysis was first conducted through hierarchical clustering. As shown in Fig. 6A, a total of 296 samples were finally screened out among all the outlier samples, which had a distance of larger than 80,000. Subsequently, the weight co-expression network was constructed by WGCNA with *β*=8 to guarantee the scale-free network (Figs. 6B and 6C). Afterwards, dynamic shear method (Dong & Horvath, 2007) was utilized to determine the gene modules, and clustering analysis was performed on these modules. Additionally, modules with close distance were further merged into the new module, having height, deepSplit and minModuleSize set to 0.25, 2 and 30, respectively. Finally, a total of 30 modules were acquired (Fig. 6D). Of note, the grey module indicated gene sets that could not be clustered into other modules. The transcripts of each module were counted and displayed in Table 1. In total, 6,679 transcripts were allocated to 29 co-expression modules. The correlations of the eigenvectors of these 30 modules with ImmuneScore, StromalScore and ESTIMATEScore were subsequently calculated, respectively. As shown in Fig. 6E, the yellow module obviously harbored extremely high association with these three immune-related scores based on ESTIMATE algorithm containing 422 genes.

**Table 1:** Number of transcripts in each module.

Modules	Genes
Black	232
Blue	676
Brown	469
Cyan	82
Darkgreen	53
Darkgrey	44
Darkorange	38
Darkred	54
Darkturquoise	47
Green	276
Greenyellow	110
Grey	7,417
Grey 60	67
Lightcyan	68
Lightgreen	66
Lightyellow	65
Magenta	181
Midnightblue	78
Orange	40
Pink	232
Purple	116
Red	261
Royalblue	64
Saddlebrown	31
Salmon	97
Skyblue	33
Tan	98
Turquoise	2,642
White	37
Yellow	422

**Figure 7:**
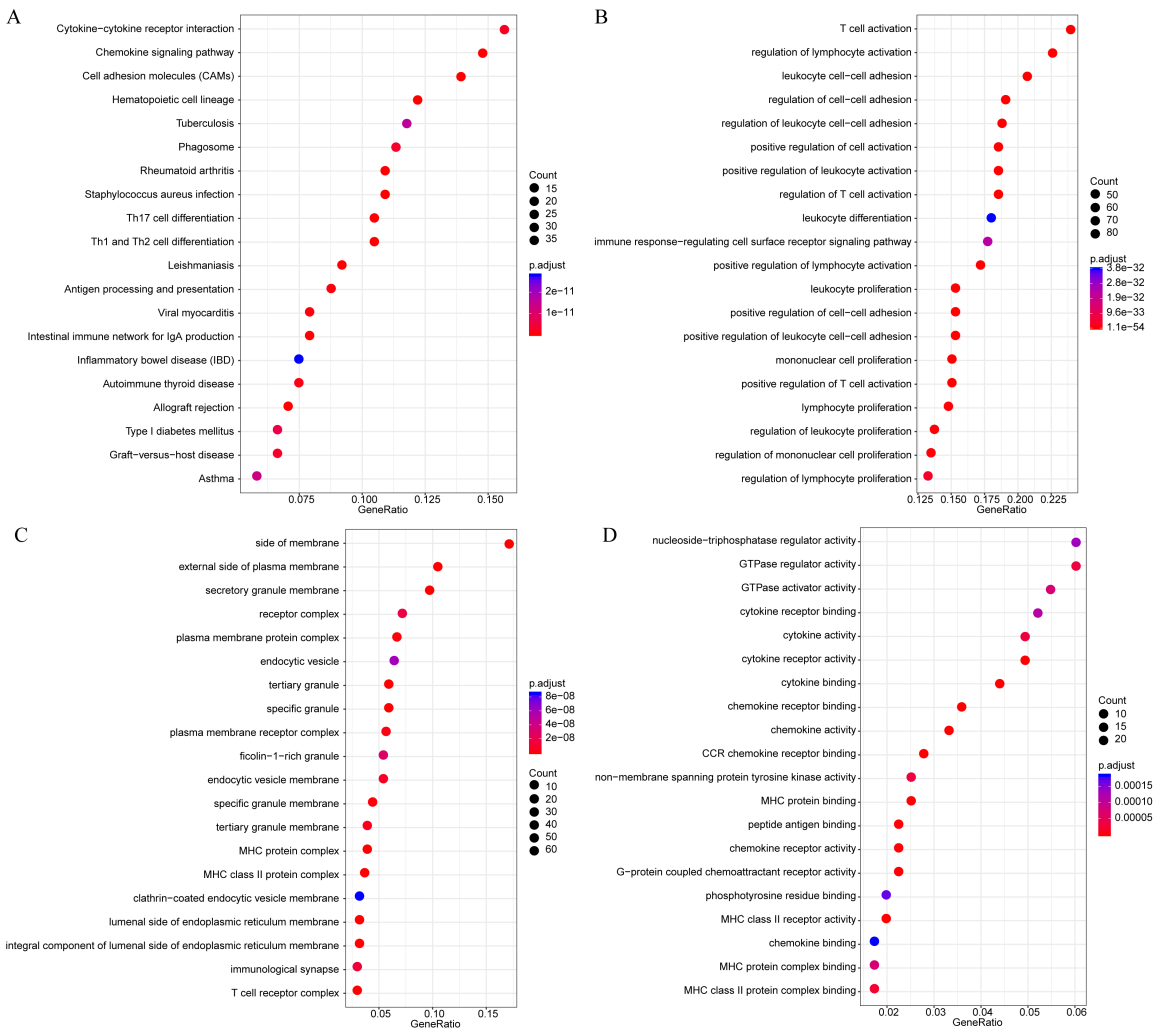
The KEGG pathway and GO enrichment analysis of the genes in yellow module. (A) Top 20 KEGG pathways enriched by the genes in yellow module. (B) Top 20 GO BP terms enriched by the genes in yellow module. (C) Top 20 GO CC terms enriched by the genes in yellow module. (D) Top 20 GO MF terms enriched by the genes in yellow module. GO, Gene Ontology; BP: biological process; CC, cellular component; MF, molecular function.

The gene functions in the yellow module were subsequently analyzed. Meanwhile, KEGG and GO enrichment analysis was also conducted using the clusterProfiler of R software package, with flase discovery rate (FDR) set as <0.05. The detailed enrichment results were shown as supporting information file (yellow enrich.txt). As a result, the genes in the yellow module were enriched into 50 KEGG pathways, 670 GO biological processes (BP), 85 GO cellular components (CC) and 74 molecular functions (MF). The most significant top 20 KEGG pathways and GO terms were shown in Fig. 7. The enriched pathways mainly included Th1 and Th2 cell differentiation, cytokine-cytokine receptor interaction and so on. And the enriched biological processes primarily included T cell activation, leukocyte cell–cell adhesion and so on. The enriched cell components mainly included MHC class II protein complex and T cell receptor complex, and so on. The enriched molecular functions mainly included cytokine receptor activity and MHC class II receptor activity, and the rest. Intriguingly, these enriched pathways and GO term have previously been reported to have close association with CSCC and its immune microenvironment (Roca et al., 2019; Wang et al., 2017; Yasmeen et al., 2010; Zehbe et al., 2005).

**Figure 8:**
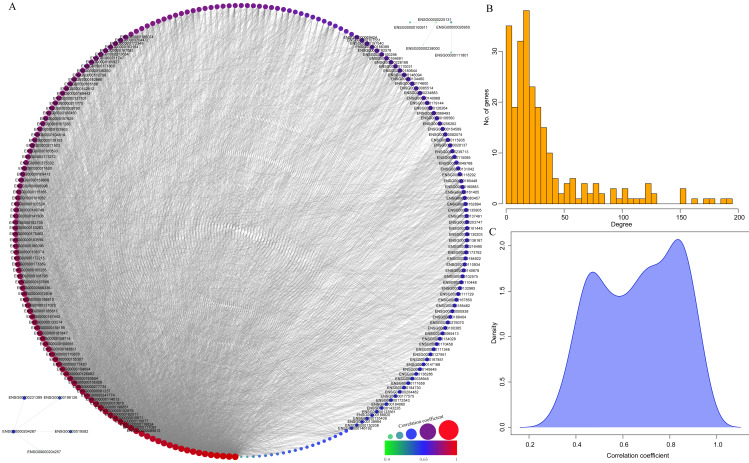
Construction of co-expression network of yellow module-related genes. (A) Co-expression network of weights between genes in yellow module. (B) The degree distribution of nodes in yellow module. (C) The correlation of genes and module in the network.

Finally, to further mine the immune scores-related genes, the weight co-expression relationship between genes in the yellow modules was calculated, with the weight threshold greater than 0.2. Cytoscape software was used for derivation and visualization of the co-expression network of these genes (as shown in Fig. 8A). Afterwards, we further analyzed the topological properties of the network, which contained 244 nodes and 4,083 edges, indicating that genes with greater association with modules had more close correlation with other genes in the network. As shown in Fig. 8B, the degree distribution of the network was further analyzed, suggesting that the degree of the majority of nodes was extremely small, while the degree of a few nodes was rather large, which was consistent with the characteristics of biological network. The correlation between the gene and the module was further calculated. As shown in Fig. 8C, the correlation between most genes and the module was over 0.6, suggesting a high expression similarity between the genes in the module. Moreover, a total of 26 genes (Table 2 and lst.genes.txt as Supplemental File) with a correlation over 0.9 and a degree over 50 in the network were selected, with seven members of LCK Metagenes, and one member of Co_inhibition Metagenes. Thus, 18 new representative immune microenvironment-related genes were finally screened.

**Table 2:** Genes with a correlation over 0.9 and a degree over 50 in the network.

ENSG	Symbol	corr.R	Degree	MeteGene
ENSG00000015285	WAS	0.964019	188	
ENSG00000110324	IL10RA	0.944217	154	LCK
ENSG00000134516	DOCK2	0.932541	113	
ENSG00000149781	FERMT3	0.957826	171	
ENSG00000043462	LCP2	0.941048	102	CLK
ENSG00000185862	EVI2B	0.94047	153	LCK
ENSG00000117091	CD48	0.918649	107	LCK
ENSG00000089012	SIRPG	0.918974	119	
ENSG00000135077	HAVCR2	0.932432	95	Co_inhibition
ENSG00000116824	CD2	0.915954	124	LCK
ENSG00000142347	MYO1F	0.962694	193	
ENSG00000198851	CD3E	0.90917	130	
ENSG00000123329	ARHGAP9	0.925285	126	
ENSG00000010671	BTK	0.913087	85	
ENSG00000105122	RASAL3	0.92036	124	
ENSG00000162511	LAPTM5	0.912211	72	
ENSG00000005844	ITGL	0.92691	125	
ENSG00000010610	CD48	0.908066	57	
ENSG00000123338	NCKAP1L	0.953232	162	
ENSG00000102879	CORO1A	0.909449	94	LCK
ENSG00000126860	EVI2A	0.923238	70	
ENSG00000143119	CD53	0.957049	178	LCK
ENSG00000160791	CCR5	0.926682	104	
ENSG00000110077	MS4A6A	0.915621	57	
ENSG00000122122	SASH3	0.949558	153	
ENSG00000167208	SNX20	0.942276	123	

### Function analysis of 18 novel representative immune microenvironment-related genes in CSCC patients

**Figure 9:**
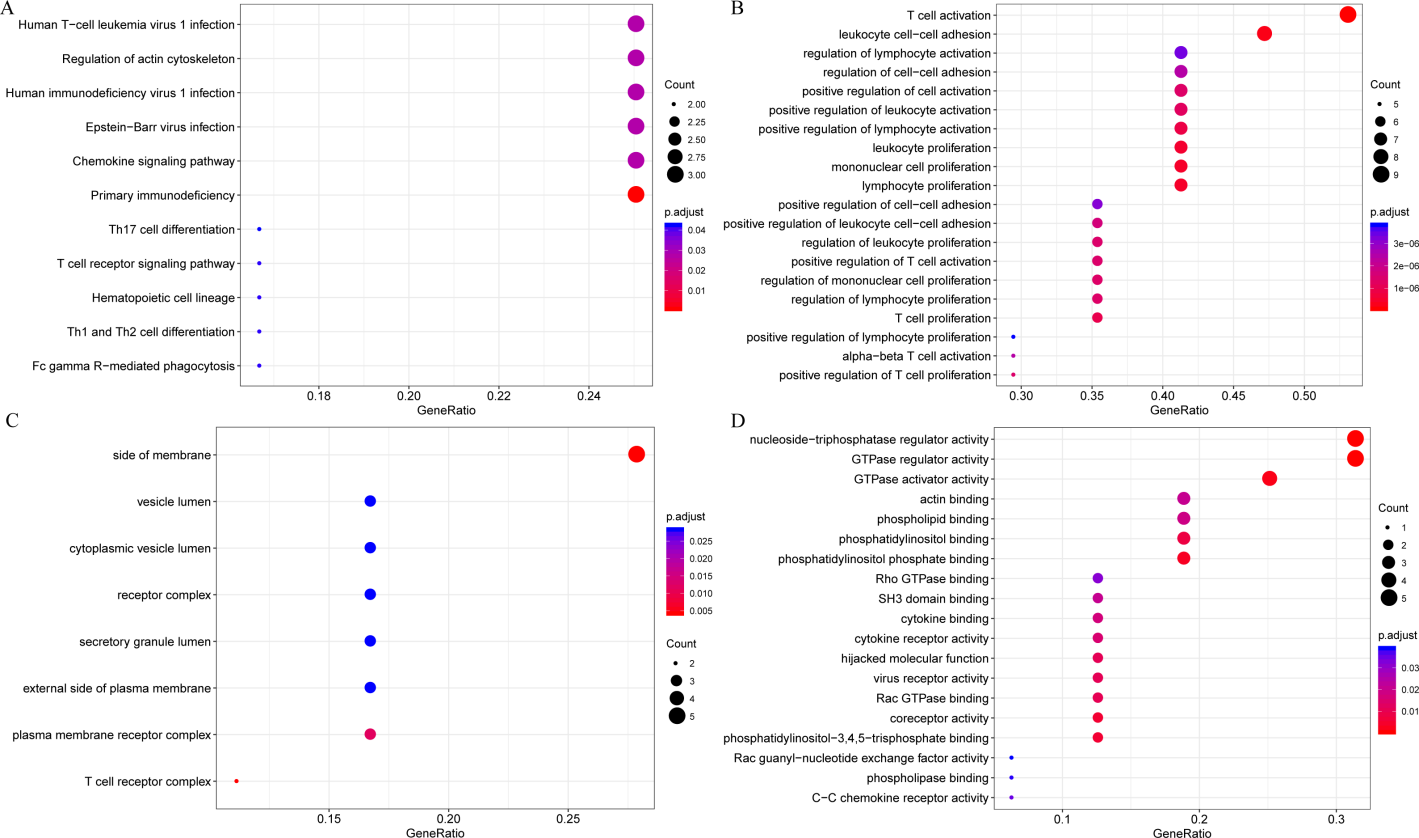
The KEGG pathway and GO enrichment analysis of 18 novel representative immune microenvironment-related genes for CSCC patients. (A) Top 20 KEGG pathways enriched by 18 novel representative immune microenvironment-related genes. (B) Top 20 GO BP terms enriched by 18 novel representative immune microenvironment-related genes. (C) Top 20 GO CC terms enriched by 18 novel representative immune microenvironment-related genes. (D) Top 20 GO MF terms enriched by 18 novel representative immune microenvironment-related genes. GO, Gene Ontology; BP, biological process; CC, cellular component; MF, molecular function.

Firstly, to further analyze the functions of these 18 novel representative immune microenvironment-related genes, the R software package clusterProfiler was utilized for KEGG and GO enrichment analysis, with the significance FDR set at <0.05. The detailed results were summarized in lst enrich.txt (Supplemental File). In brief, these 18 genes were enriched into 11 KEGG pathways, 202 GO biological processes, 8 GO cell components, 19 molecular functions. The most significant 20 KEGG pathways and GO terms were shown in Fig. 9, the majority of which were involved in the proliferation, growth and differentiation of T cells. Intriguingly, LAPTM5, EVI2A and MS4A6A were not enriched in any signaling pathways and GO term, indicating that the functions of these three genes remained completely unclear, which is the focus of our further studies.

**Figure 10:**
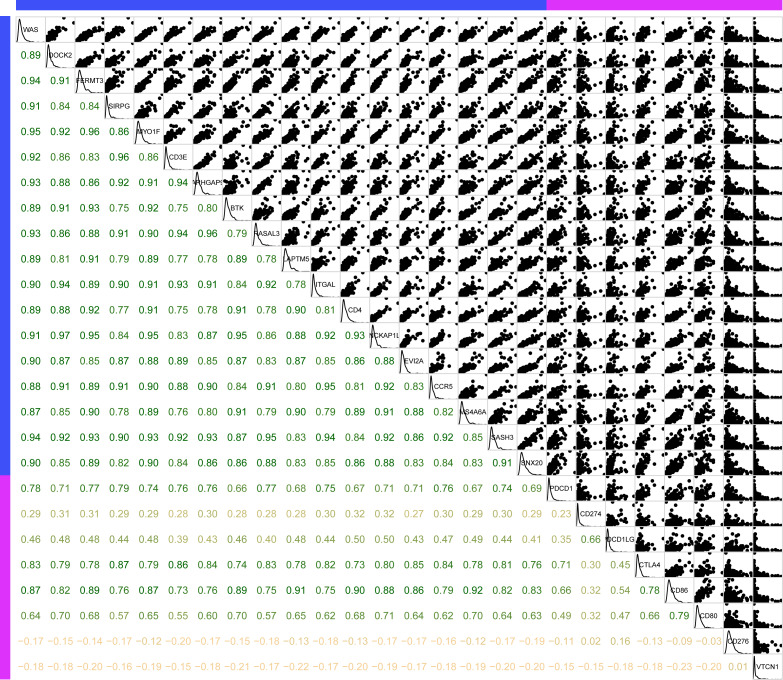
The association between 18 novel representative immune microenvironment-related genes for CSCC patients and immune checkpoints. Apart from CD276 and VTCN1, the other 6 immune checkpoints were significantly related to 18 immune microenvironment-related genes.

Secondly, to further investigate the potential roles of the 18 novel representative immune microenvironment-related genes in clinical practice, the R package corrgram was utilized for the calculation of the association between these genes and immune checkpoints (PDCD1, CD274, PDCD1LG2, CTLA4, CD86, CD80, CD276, VTCN1). As shown in Fig. 10, apart from CD276 and VTCN1, the other 6 immune checkpoints were significantly related to these 18 genes, with an average correlation coefficient over 0.5, which indicated that these immune microenvironment-related genes might be promising targets for immunotherapy.

**Figure 11:**
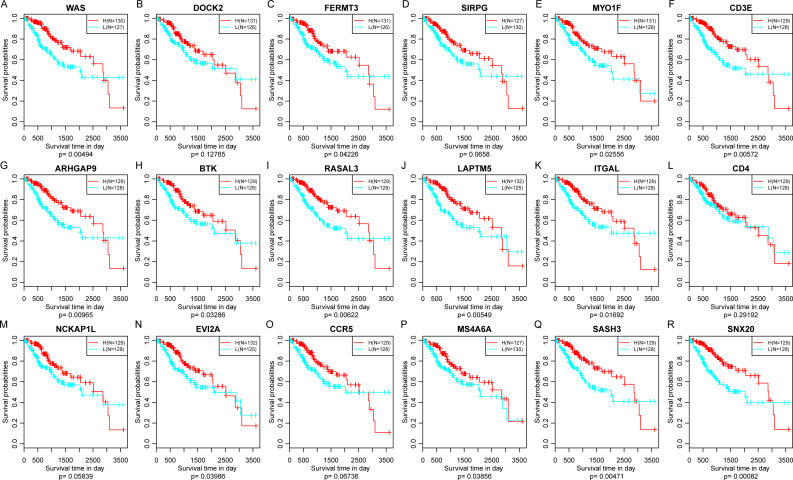
The relationship between 18 novel representative immune microenvironment-related genes and prognosis (A–R). Apart from DOCK2 (*P* = 0.12765), SIRPG (*P* = 0.0658), CD4 (*P* = 0.29192), NCKAP1L (*P* = 0.12765) and CCR5 (*P* = 0.06736), high expression of other 13 genes were significantly associated with better overall survival.

Finally, the prognostic significance of 18 novel representative immune microenvironment-related genes was assessed. According to the median of gene expression, samples were categorized into high and low expression groups. And then the differences of prognosis between these groups were analyzed. As shown in Fig. 11, high expression of 13 genes were significantly associated with better overall survival according to the threshold of *p* < 0.05, suggesting that these genes might be closely associated with patient prognosis.

### Validation of the correlations of 18 immune microenvironment-related genes with ImmuneScore for CSCC patients by using external dataset

**Figure 12:**
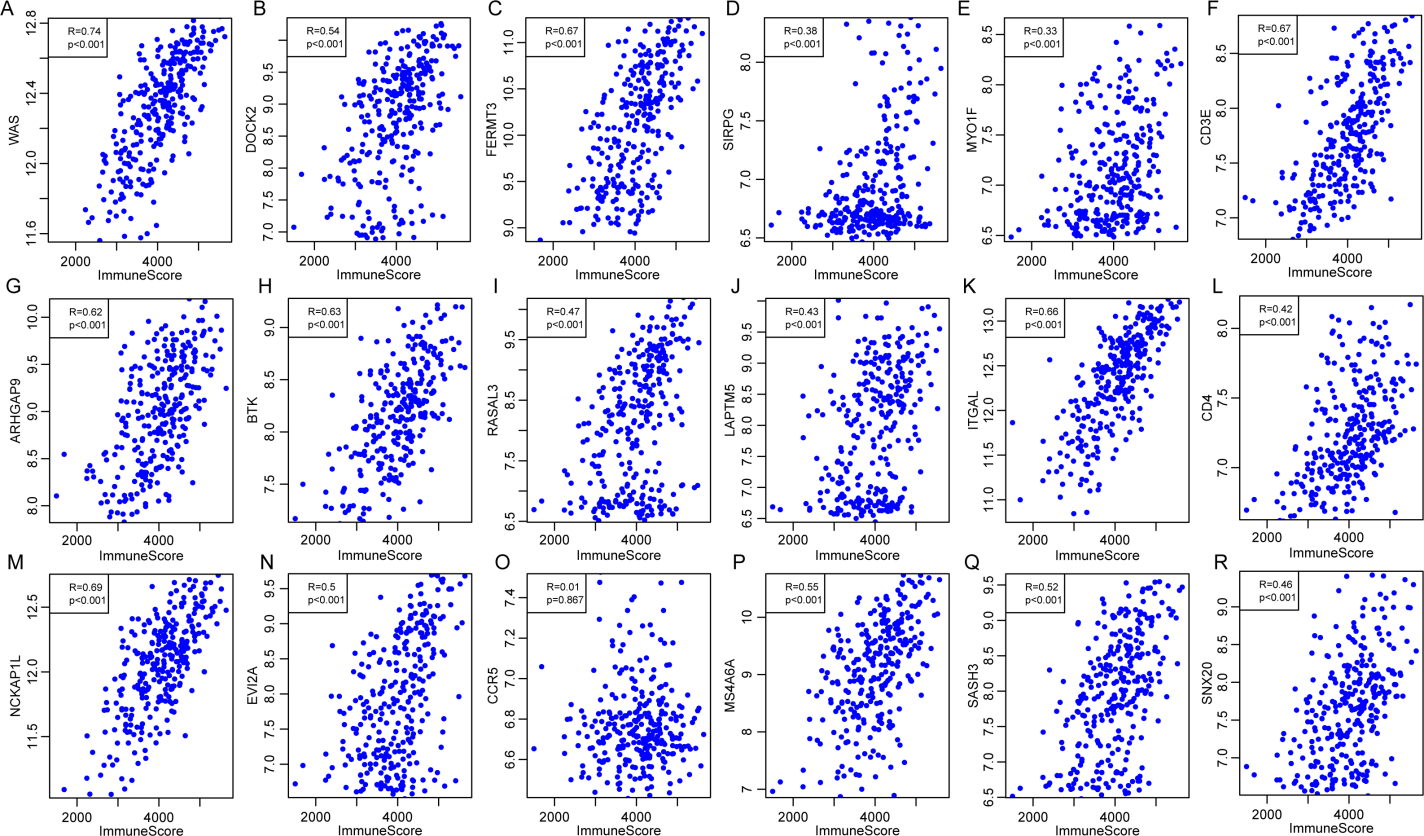
The correlations of 18 immune microenvironment-related genes with ImmuneScore for CSCC patients in independent dataset (A–R). Apart from CCR5 (*P* = 0.867, *R* = 0.01), the other 17 genes were significantly associated with the ImmuneScore.

External database was used for further validation of the correlations of 18 immune microenvironment-related genes with the immune-related scores according to ESTIMATE algorithm for CSCC patients. Standardized expression matrix was downloaded and extracted from an independent dataset GSE44001 (Lee et al., 2013) from Gene Expression Omnibus (GEO). R packages hgu133plus2.db was utilized to map a probe for gene to extract the expression profiles of these 18 genes, followed by the calculation of the ImmuneScore for each sample using R software package ESTIMATE. Subsequently, the Pearson correlation was calculated between expression of these genes and the level of ImmuneScore for every CSCC sample in this dataset. As shown in Fig. 12, apart from CCR5 (*P* = 0.867, *R* = 0.01), the other 17 genes were significantly associated with the ImmuneScore, which was consistent with our previous findings.

## Discussion

Great attention has been paid to the association of the immune system with the pathogenesis and progression of tumor in recent years, which has shed light on CSCC therapy, promoting the continuous development of anti-cancer therapy (Dyer et al., 2019; Orbegoso, Murali & Banerjee, 2018). The external anti-CSCC approaches are frequently applied in previous clinical practice, including surgical resection and chemotherapy. However, the effect of surgical resection is generally restricted due to the invasion into adjacent tissues by cancer cells or distant metastasis. In addition, the application of chemotherapy is limited due to its toxicity to normal tissues (Menderes et al., 2016). Thus, conventional therapies would exert great burden on the body while providing therapeutic benefits. To this end, it has been widely accepted as a novel direction of anti-cancer therapy by starting from the tumor origin, in other words, the immune system of human body, to control and even kill tumor cells via the modulation of the immune system and enhancement of the anti-tumor immunity in the tumor microenvironment (Ring et al., 2017).

The tumor microenvironment, mainly composed of immune cells, inflammatory cells, mesenchymal cells, tumor cells, stromal cells, inflammatory mediators and cytokines, provides support for tumor biological behavior including the pathogenesis, progression, invasion and metastasis (Piersma, 2011; Qi & Wu, 2019; Tuccitto et al., 2019). Therefore, it is of great significance to discover novel and meaningful immune microenvironment-related genes in CSCC as prognostic predictor and therapeutic targets.

In this study, the TCGA database was used to search for the immune microenvironment markers related to the survival time of CSCC patients. And 18 genes were finally detected having remarkable correlation with the prognosis of patients, which was further validated in the GEO database.

To be specific, firstly, multiple methods of data analysis were utilized to search for the three immune-related scores on the basis of ESTIMATE algorithm, showing high correlations with diverse other immune-related scores, patients prognosis, HPV infection status and the mutation levels of multiple well-defined CSCC-related genes (HLA and TP53). Secondly, the representative genes in the gene modules associated with immune-related scores according to ESTIMATE algorithm were further searched using WGCNA and network topology analysis. Thirdly, we mined the gene functions through enrichment analysis, followed by the exploration of the association between these genes and immune checkpoint genes. Finally, survival analysis was employed to search for the genes with evident correlation with OS. In addition, external database was employed for further validation of the association of these immune microenvironment-related genes with ImmuneScore for CSCC patients. In total, we successfully mined 18 novel potential immune microenvironment-related diagnostic and prognostic indicators or therapeutic targets.

Of note, 11 out of these 18 genes (IL10RA, CD4, HAVCR2, CD2, CCR5, CD3E, BTK, etc.) have previously been demonstrated to participate in the pathogenesis, progression, malignant transformation, and pathological process of immune microenvironment of CSCC, which are also significantly associated with patient survival, prognosis and diagnosis (Cao et al., 2013; Che, Shao & Wang, 2016; Hussain et al., 2013; Punt et al., 2015). These above-described observations validate the great reliability and accuracy of the bioinformatic mining results in our present study, in which, we combined TCGA database screening with GEO database for verification. However, the correlations of two genes (LAPTM5 and EVI2A) with CSCC have never been confirmed by any basic or clinical studies, which we are most interested in. LAPTM5, Laptm5, a lysosomal transmembrane protein enhancing the degradation of several targets involved in immune signaling (such as ubiquitin-editing enzyme A20), has been validated to be participate in the modulation of the lethal T cell alloreactivity mediated by dendritic cells and immunoreactions in multiple inflammatory disease, such as host versus graft disease (GVHD) (Glowacka et al., 2012; Hubbard-Lucey et al., 2014). On the other hand, EVI2A has been confirmed to be involved in lymphocyte proliferation and viability, which is a well-defined immune-specific tumor suppressor in head and neck cancer (Li et al., 2014).

At present, accumulating studies focus on the mining of the association of numerous genes expression with the survival of CSCC patients, however, the majority of previous studies are only performed in animal model, in vitro cell model or small sample samples of tumor patients. Thus, more comprehensive, large-scale population studies are required due to the complexity of CSCC microenvironment. Fortunately, the rapid development of genome-wide sequencing renders the free utilization of high-throughput tumor databases, such as TCGA, making it possible to apply the bioinformatic big data for the large-scale CSCC population.

## Conclusion

In the present study, we mainly studied the CSCC immune microenvironment-related gene characteristics. Consequently, these genes are involved in the pathogenesis, progression and malignant transformation of CSCC, affecting OS of CSCC patients. Our present findings can offer more information to decode the complex tumor-tumor interactions in CSCC microenvironment. These findings will help to mine the novel immune-related diagnostic indicators, therapeutic targets and prognostic predictors in CSCC. Besides, the methods of our study have general applicability and provide some references value for the identification of potential diagnostic and prognostic biomarkers for other biologically heterogeneous cancers.

##  Supplemental Information

10.7717/peerj.9627/supp-1 Supplemental Information 1 The ImmuneScore, StromalScore and ESTIMATEScore of each sample

10.7717/peerj.9627/supp-2 Supplemental Information 2 The scores of each sample in the immunocyte infiltration

10.7717/peerj.9627/supp-3 Supplemental Information 3 Score of the abundances of ten immune-related cell populations in the tumor microenvironment

10.7717/peerj.9627/supp-4 Supplemental Information 4 The scores of each sample in the 13 types of metagenes

10.7717/peerj.9627/supp-5 Supplemental Information 5 13 metagenes corresponded to various immunocyte types

10.7717/peerj.9627/supp-6 Supplemental Information 6 KEGG and GO enrichment analysis results of 18 novel representative immune microenvironment-related genes

10.7717/peerj.9627/supp-7 Supplemental Information 7 26 genes with a correlation over 0.9 and a degree over 50 in the network

10.7717/peerj.9627/supp-8 Supplemental Information 8 The enrichment outcomes of yellow module

## Additional Information and Declarations

### Data Availability

The following information was supplied regarding data availability: The raw measurements are available as Supplemental Files.

## References

[ref-1] Altobelli E, Rapacchietta L, Profeta VF, Fagnano R (2019). HPV-vaccination and cancer cervical screening in 53 WHO European Countries: an update on prevention programs according to income level. Cancer Medicine.

[ref-2] Alvarado-Cabrero I, Roma AA, Park KJ, Rutgers JKL, Silva EG (2017). Factors predicting pelvic lymph node metastasis, relapse, and disease outcome in pattern C endocervical adenocarcinomas. International Journal of Gynecological Pathology.

[ref-3] Angeles MA, Baissas P, Leblanc E, Lusque A, Ferron G, Ducassou A, Martinez-Gomez C, Querleu D, Martinez A (2019). Magnetic resonance imaging after external beam radiotherapy and concurrent chemotherapy for locally advanced cervical cancer helps to identify patients at risk of recurrence. International Journal of Gynecological Cancer.

[ref-4] Bachtiary B, Boutros PC, Pintilie M, Shi W, Bastianutto C, Li JH, Schwock J, Zhang W, Penn LZ, Jurisica I, Fyles A, Liu FF (2006). Gene expression profiling in cervical cancer: an exploration of intratumor heterogeneity. Clinical Cancer Research.

[ref-5] Bosque MAS, Cervantes-Bonilla MA, Palacios-Saucedo GDC (2018). Clinical and dosimetric factors associated with the development of hematologic toxicity in locally advanced cervical cancer treated with chemotherapy and 3D conformal radiotherapy. Reports of Practical Oncology and Radiotherapy.

[ref-6] Cao Y, Zhou X, Huang X, Li Q, Gao L, Jiang L, Huang M, Zhou J (2013). Tim-3 expression in cervical cancer promotes tumor metastasis. PLOS ONE.

[ref-7] Che LF, Shao SF, Wang LX (2016). Downregulation of CCR5 inhibits the proliferation and invasion of cervical cancer cells and is regulated by microRNA-107. Experimental and Therapeutic Medicine.

[ref-8] Chen SB, Yang XH, Weng HR, Liu DT, Li H, Chen YP (2017). Clinicopathological features and surgical treatment of cervical oesophageal cancer. Scientific Reports.

[ref-9] Chen XJ, Wu S, Yan RM, Fan LS, Yu L, Zhang YM, Wei WF, Zhou CF, Wu XG, Zhong M, Yu YH, Liang L, Wang W (2019). The role of the hypoxia-Nrp-1 axis in the activation of M2-like tumor-associated macrophages in the tumor microenvironment of cervical cancer. Molecular Carcinogenesis.

[ref-10] Cheng W, Ren X, Zhang C, Cai J, Liu Y, Han S, Wu A (2016). Bioinformatic profiling identifies an immune-related risk signature for glioblastoma. Neurology.

[ref-11] De Nola R, Menga A, Castegna A, Loizzi V, Ranieri G, Cicinelli E, Cormio G (2019). The crowded crosstalk between cancer cells and stromal microenvironment in gynecological malignancies: biological pathways and therapeutic implication. International Journal of Molecular Sciences.

[ref-12] Ding L, Liu C, Zhou Q, Feng M, Wang J (2019). Association of estradiol and HPV/HPV16 infection with the occurrence of cervical squamous cell carcinoma. Oncology Letters.

[ref-13] Dong J, Horvath S (2007). Understanding network concepts in modules. BMC Systems Biology.

[ref-14] Dyer BA, Zamarin D, Eskandar RN, Mayadev JM (2019). Role of immunotherapy in the management of locally advanced and recurrent/metastatic cervical cancer. Journal of the National Comprehensive Cancer Network.

[ref-15] Fest J, Ruiter R, Mulder M, Groot Koerkamp B, Ikram MA, Stricker BH, Van Eijck CHJ (2019). The systemic immune-inflammation index is associated with an increased risk of incident cancer—a population-based cohort study. International Journal of Cancer.

[ref-16] Gil-Moreno A, Magrina JF (2019). Minimally invasive or abdominal radical hysterectomy for cervical cancer. New England Journal of Medicine.

[ref-17] Glowacka WK, Alberts P, Ouchida R, Wang JY, Rotin D (2012). LAPTM5 protein is a positive regulator of proinflammatory signaling pathways in macrophages. Journal of Biological Chemistry.

[ref-18] Guo W, Cai S, Zhang F, Shao F, Zhang G, Zhou Y, Zhao L, Tan F, Gao S, He J (2019). Systemic immune-inflammation index (SII) is useful to predict survival outcomes in patients with surgically resected non-small cell lung cancer. Thorac Cancer.

[ref-19] Heintzelman DL, Lotan R, Richards-Kortum RR (2000). Characterization of the autofluorescence of polymorphonuclear leukocytes, mononuclear leukocytes and cervical epithelial cancer cells for improved spectroscopic discrimination of inflammation from dysplasia. Photochemistry and Photobiology.

[ref-20] Huang H, Liu Q, Zhu L, Zhang Y, Lu X, Wu Y, Liu L (2019). Prognostic value of preoperative systemic immune-inflammation index in patients with cervical cancer. Scientific Reports.

[ref-21] Hubbard-Lucey VM, Shono Y, Maurer K, West ML, Singer NV, Ziegler CG, Lezcano C, Motta AC, Schmid K, Levi SM, Murphy GF, Liu C, Winkler JD, Amaravadi RK, Rogler G, Dickinson AM, Holler E, Van den Brink MR, Cadwell K (2014). Autophagy gene Atg16L1 prevents lethal T cell alloreactivity mediated by dendritic cells. Immunity.

[ref-22] Hussain SK, Madeleine MM, Johnson LG, Du Q, Galloway DA, Daling JR, Malkki M, Petersdorf EW, Schwartz SM (2013). Nucleotide variation in IL-10 and IL-12 and their receptors and cervical and vulvar cancer risk: a hybrid case-parent triad and case-control study. International Journal of Cancer.

[ref-23] Ishibashi Y, Tsujimoto H, Hiraki S, Kumano I, Yaguchi Y, Horiguchi H, Nomura S, Ito N, Shinto E, Aosasa S, Yamamoto J, Ueno H (2018). Prognostic value of preoperative systemic immunoinflammatory measures in patients with esophageal cancer. Annals of Surgical Oncology.

[ref-24] Kidd EA, Grigsby PW (2008). Intratumoral metabolic heterogeneity of cervical cancer. Clinical Cancer Research.

[ref-25] Laprano TD, Lemos EH, Cunha LM, Junior JE, De SousaTeles RA, Rabenhorst SH (2014). Association of TP53 codon 72 and intron 3 16-bp Ins/Del polymorphisms with cervical cancer risk. Tumour Biology.

[ref-26] Lee YY, Choi CH, Sung CO, Do IG, Hub SJ, Kim HJ, Kim TJ, Lee JW, Bae DS, Kim BG (2012). Clinical significance of changes in peripheral lymphocyte count after surgery in early cervical cancer. Gynecologic Oncology.

[ref-27] Lee YY, Kim TJ, Kim JY, Choi CH, Do IG, Song SY, Sohn I, Jung SH, Bae DS, Lee JW, Kim BG (2013). Genetic profiling to predict recurrence of early cervical cancer. Gynecologic Oncology.

[ref-28] Li B, Severson E, Pignon JC, Zhao H, Li T, Novak J, Jiang P, Shen H, Aster JC, Rodig S, Signoretti S, Liu JS, Liu XS (2016). Comprehensive analyses of tumor immunity: implications for cancer immunotherapy. Genome Biology.

[ref-29] Li B, Wang X, Chen H, Shang LX, Wu N (2015). TP53 codon 72 polymorphism and susceptibility to cervical cancer in the Chinese population: an update meta-analysis. International Journal of Clinical and Experimental Medicine.

[ref-30] Li F, Guo H, Wang Y, Liu B, Zhou H (2020). Profiles of tumor-infiltrating immune cells and prognostic genes associated with the microenvironment of bladder cancer. International Immunopharmacology.

[ref-31] Li XW, Rees JS, Xue P, Zhang H, Hamaia SW, Sanderson B, Funk PE, Farndale RW, Lilley KS, Perrett S, Jackson AP (2014). New insights into the DT40 B cell receptor cluster using a proteomic proximity labeling assay. Journal of Biological Chemistry.

[ref-32] Lu G, Chen L, Wu S, Feng Y, Lin T (2020). Comprehensive analysis of tumor-infiltrating immune cells and relevant therapeutic strategy in esophageal cancer. Disease Markers.

[ref-33] Lu XY, Chen M, Chen DH, Li Y, Liu PT, Liu Y (2018). Remifentanil on T lymphocytes, cognitive function and inflammatory cytokines of patients undergoing radical surgery for cervical cancer. European Review for Medical and Pharmacological Sciences.

[ref-34] Marth C, Landoni F, Mahner S, McCormack M, Gonzalez-Martin A, Colombo N, ESMO Guidelines Committee (2018). Cervical cancer: ESMO Clinical Practice Guidelines for diagnosis, treatment and follow-up. Annals of Oncology.

[ref-35] Matsuo K, Machida H, Mandelbaum RS, Konishi I, Mikami M (2019). Validation of the 2018 FIGO cervical cancer staging system. Gynecologic Oncology.

[ref-36] Menderes G, Black J, Schwab CL, Santin AD (2016). Immunotherapy and targeted therapy for cervical cancer: an update. Expert Review of Anticancer Therapy.

[ref-37] Minion LE, Tewari KS (2018). Cervical cancer—state of the science: from angiogenesis blockade to checkpoint inhibition. Gynecologic Oncology.

[ref-38] Nanthamongkolkul K, Hanprasertpong J (2018). Predictive factors of pelvic lymph node metastasis in early-stage cervical cancer. Oncology Research and Treatment.

[ref-39] Ngo-Metzger Q, Adsul P (2019). Screening for cervical cancer. American Family Physician.

[ref-40] Orbegoso C, Murali K, Banerjee S (2018). The current status of immunotherapy for cervical cancer. Reports of Practical Oncology and Radiotherapy.

[ref-41] Otter SJ, Chatterjee J, Stewart AJ, Michael A (2019). The role of biomarkers for the prediction of response to checkpoint immunotherapy and the rationale for the use of checkpoint immunotherapy in cervical cancer. Clinical Oncology Journal.

[ref-42] Piersma SJ (2011). Immunosuppressive tumor microenvironment in cervical cancer patients. Cancer Microenvironment.

[ref-43] Punt S, Houwing-Duistermaat JJ, Schulkens IA, Thijssen VL, Osse EM, De Kroon CD, Griffioen AW, Fleuren GJ, Gorter A, Jordanova ES (2015). Correlations between immune response and vascularization qRT-PCR gene expression clusters in squamous cervical cancer. Molecular Cancer.

[ref-44] Qi D, Wu E (2019). Cancer prognosis: considering tumor and its microenvironment as a whole. EBioMedicine.

[ref-45] Rangel-Corona R, Corona-Ortega T, Del Rio-Ortiz I, Nieves-Ramirez ME, Moran-Banuelos H, Gonzalez-Tenorio O, Caceres-Cortes JR, Weiss-Steider B (2011). Cationic liposomes bearing IL-2 on their external surface induced mice leukocytes to kill human cervical cancer cells in vitro, and significantly reduced tumor burden in immunodepressed mice. Journal of Drug Targeting.

[ref-46] Ring KL, Yemelyanova AV, Soliman PT, Frumovitz MM, Jazaeri AA (2017). Potential immunotherapy targets in recurrent cervical cancer. Gynecologic Oncology.

[ref-47] Roca AM, Chobrutskiy BI, Callahan BM, Blanck G (2019). T-cell receptor V and J usage paired with specific HLA alleles associates with distinct cervical cancer survival rates. Human Immunology.

[ref-48] Safonov A, Jiang T, Bianchini G, Gyorffy B, Karn T, Hatzis C, Pusztai L (2017). Immune gene expression is associated with genomic aberrations in breast cancer. Cancer Research.

[ref-49] Shannon P, Markiel A, Ozier O, Baliga NS, Wang JT, Ramage D, Amin N, Schwikowski B, Ideker T (2003). Cytoscape: a software environment for integrated models of biomolecular interaction networks. Genome Research.

[ref-50] Shen L, Yu H, Liu M, Wei D, Liu W, Li C, Chang Q (2018). A ten-long non-coding RNA signature for predicting prognosis of patients with cervical cancer. OncoTargets and Therapy.

[ref-51] Srivastava S, Koay EJ, Borowsky AD, De Marzo AM, Ghosh S, Wagner PD, Kramer BS (2019). Cancer overdiagnosis: a biological challenge and clinical dilemma. Nature Reviews Cancer.

[ref-52] Szklarczyk D, Gable AL, Lyon D, Junge A, Wyder S, Huerta-Cepas J, Simonovic M, Doncheva NT, Morris JH, Bork P, Jensen LJ, Mering CV (2019). STRING v11: protein-protein association networks with increased coverage, supporting functional discovery in genome-wide experimental datasets. Nucleic Acids Research.

[ref-53] Tuccitto A, Shahaj E, Vergani E, Ferro S, Huber V, Rodolfo M, Castelli C, Rivoltini L, Vallacchi V (2019). Immunosuppressive circuits in tumor microenvironment and their influence on cancer treatment efficacy. Virchows Archiv.

[ref-54] Uppal S, Rebecca Liu J, Kevin Reynolds R, Rice LW, Spencer RJ (2019). Trends and comparative effectiveness of inpatient radical hysterectomy for cervical cancer in the United States (2012-2015). Gynecologic Oncology.

[ref-55] Wang X, Jiang Y, Yuan M, Chen C, Wang K, Zhang Q, Zuo Y, Ren S (2017). Overexpression of dendritic cell-specific intercellular adhesion molecule-3-grabbing nonintegrin-related protein in cervical cancer and correlation with squamous cell carcinoma antigen. Oncology Letters.

[ref-56] Xia WX, Yu Q, Li GH, Liu YW, Xiao FH, Yang LQ, Rahman ZU, Wang HT, Kong QP (2019). Identification of four hub genes associated with adrenocortical carcinoma progression by WGCNA. PeerJ.

[ref-57] Xiao X, Liu L, Li WJ, Liu J, Chen DJ (2013). HLA-A, HLA-B, HLA-DRB1 polymorphisms and risk of cervical squamous epithelial cell carcinoma: a population study in China. Asian Pacific Journal of Cancer Prevention.

[ref-58] Xie QK, Chen P, Hu WM, Sun P, He WZ, Jiang C, Kong PF, Liu SS, Chen HT, Yang YZ, Wang D, Yang L, Xia LP (2018). The systemic immune-inflammation index is an independent predictor of survival for metastatic colorectal cancer and its association with the lymphocytic response to the tumor. Journal of Translational Medicine.

[ref-59] Xu Z, Wang C, Xiang X, Li J, Huang J (2019). Characterization of mRNA expression and endogenous RNA profiles in bladder cancer based on the cancer genome atlas (TCGA) database. Medical Science Monitor.

[ref-60] Yang S, Wu Y, Deng Y, Zhou L, Yang P, Zheng Y, Zhang D, Zhai Z, Li N, Hao Q, Song D, Kang H, Dai Z (2019). Identification of a prognostic immune signature for cervical cancer to predict survival and response to immune checkpoint inhibitors. Oncoimmunology.

[ref-61] Yasmeen A, Alachkar A, Dekhil H, Gambacorti-Passerini C, Al Moustafa AE (2010). Locking Src/Abl tyrosine kinase activities regulate cell differentiation and invasion of human cervical cancer cells expressing E6/E7 oncoproteins of high-risk HPV. International Journal of Oncology.

[ref-62] Yoshihara K, Shahmoradgoli M, Martinez E, Vegesna R, Kim H, Torres-Garcia W, Trevino V, Shen H, Laird PW, Levine DA, Carter SL, Getz G, Stemke-Hale K, Mills GB, Verhaak RG (2013). Inferring tumour purity and stromal and immune cell admixture from expression data. Nature Communications.

[ref-63] Zehbe I, Hohn H, Pilch H, Neukirch C, Freitag K, Maeurer MJ (2005). Differential MHC class II component expression in HPV-positive cervical cancer cells: implication for immune surveillance. International Journal of Cancer.

[ref-64] Zhang Y, Ma D, Zhang Y, Tian Y, Wang X, Qiao Y, Cui B (2011). The imbalance of Th17/Treg in patients with uterine cervical cancer. Clinica Chimica Acta.

[ref-65] Zou KH, O’Malley AJ, Mauri L (2007). Receiver-operating characteristic analysis for evaluating diagnostic tests and predictive models. Circulation.

